# Doctors’ Bills Must Be Paid

**Published:** 1897-06

**Authors:** 


### DOCTORS' BILLS MUST BE PAID.

The New York legislature has under consideration a bill providing for the payments of debts, as follows: Every executor and administrator must proceed with diligence to pay the debts of the deceased according to the following order: 1. Debts entitled to a preference under the laws of the United States. 2. Taxes assessed on the property of the deceased previous to his death. 3. Debts of the deceased, because of services rendered and materials furnished by physicians, pharmacists, nurses and undertakers. 4. Judgments docketed, and decrees entered against the deceased according to the priority thereof respectively. 5. All recognizances, bonds, sealed instruments, notes, bills, and unliquidated demands and accounts. Preference shall not be given in the payment of a debt over the other debts of the same class, except those specified in the fourth class. All debts specified in the third class shall become due upon the death of the deceased, and shall be paid within ninety days thereafter.—*Medical News*.

